# Comparison of Sexual Function Before and After COVID-19 Infection in Female Patients

**DOI:** 10.7759/cureus.18156

**Published:** 2021-09-21

**Authors:** Muhammad Umair Nawaz, Edgar Rivera, Sagar Vinayak, Kanwal Elahi, Manoj Kumar, Momal Chand, Sana Ezae, Dua Khalid, Sidra Naz, Faizan Shaukat

**Affiliations:** 1 Internal Medicine, Jinnah Sindh Medical University, Karachi, PAK; 2 Internal Medicine, Universidad Autónoma de Guadalajara, Zapopan, MEX; 3 Internal Medicine, American University of Barbados, Bridgetown, BRB; 4 Internal Medicine, Jinnah Post Graduate Medical Center, Karachi, PAK; 5 Internal Medicine, Dow University of Health Sciences, Karachi, PAK; 6 Pathology, Ascension St. John Hospital, Detroit, USA; 7 Internal Medicine, University of Health Sciences, Lahore, PAK; 8 Internal Medicine, Dow University of Health Sciences, Civil Hospital Karachi, Karachi, PAK

**Keywords:** women fsfi, libido, sexual function, sars-cov-2 (severe acute respiratory syndrome coronavirus -2), covid-19

## Abstract

Introduction

Ample data regarding the impact of coronavirus disease-2019 (COVID-19) on the pulmonary, nervous, and gastrointestinal systems are available. However, its impact on sexual performance is understudied. In this study, we will determine the impact of COVID-19 on the sexual performance of females.

Methods

This longitudinal study was conducted in the COVID-19 unit of a tertiary care hospital in Pakistan from June 2020 to March 2021. We enrolled 300 female patients admitted to the hospital due to severe COVID-19. Patients' female sexual function index (FSFI) scale was assessed at the time of discharge. Participants were asked to answer the question based on their sexual performance before they contracted COVID-19. They were asked to return after 60 days, where FSFI was assessed again.

Results

The mean FSFI score for participants before COVID-19 was significantly higher compared to the score 60 days after discharge (28.16 ± 1.9 vs. 24.43 ± 2.5; p-value: <0.0001). Participants who had FSFI score more than 26 were significantly higher before COVID-19 (72.5% vs. 51.0%; p-value: <0.0001).

Conclusion

There is a significant decline in sexual function of females, who had contracted COVID-19 infection. COVID-19 survivors should be counseled properly about the impact on the sexual function when discussing long-term complications of COVID-19.

## Introduction

In 2019, a highly contagious and pathogenic viral infection, coronavirus disease-2019 (COVID-19), was reported for the very first time in Wuhan, China, which led to a formidable outbreak in many of its cities, followed by a global pandemic and dramatic loss of human life worldwide [1-4]. This infection is caused by the largest group of enveloped, positive-sense RNA viruses. It has several different subtypes and disease patterns, among which acute respiratory syndrome is the most serious [5,6].

Its main mode of transmission is through the nose and mouth and occurs rapidly when in close contact with an infected person, exposed via respiratory droplets or aerosols [4,7]. The information regarding its spread led to some rigorous and unprecedented preventive measures, including academic center closures, commercial activities limitations, travel restrictions, and isolation to ensure social distancing and avoid human crowding [8]. Although these procedures served to decrease the spread of the virus, they also caused increased socioeconomic instability, global frustration, and negative implications on the mental and sexual health of the individuals [4,5].

The symptoms of COVID-19 range from mild to severe. Not only does it affect the respiratory system, but clinical studies suggest multisystem involvement of COVID-19 including neurological, gastrointestinal, and opthalmological systems. The neurological symptoms, including dizziness, headache, and sense of taste and smell impairment, are frequently reported in COVID-19 [8]. The most commonly reported opthalmological symptom is conjunctival irritation, followed by diplopia and cotton wool spots [9]. It has impacts on the gastrointestinal system as well, with diarrhea being the most common symptom [10]. While there are ample data available on how COVID-19 impacts the pulmonary, nervous, and gastrointestinal systems, its impact on sexual performance is understudied. In this study, we will determine the impact of COVID-19 on the sexual performance of females.

## Materials and methods

This longitudinal study was conducted in the COVID-19 unit of a tertiary care hospital in Pakistan from June 2020 to March 2021. We enrolled 300 female patients admitted to the hospital due to severe COVID-19. Participants with pre-existing hypertension and type 2 diabetes were excluded from the study. Similarly, participants with a body mass index of more than 25 kg/m^2^ and older than 50 years were excluded from the study. Participants were enrolled at the time of their discharge via consecutive convenient non-probability sampling. Informed consent was obtained from the participants. They were informed that their participation is voluntary and at any moment they can pull out from the study if they are not comfortable. Ethical review board approval was taken from Jinnah Sindh Medical University (JSMU/IRB/2020/26). A questionnaire was composed using the FSFI and questions were inquired in full privacy from the patients. Participants were asked to answer the question based on their sexual performance before they contracted COVID-19. They were asked to return after 60 days, where FSFI was assessed again.

A self-structured questionnaire was designed that included the patient’s characteristics such as age, gender, and history of smoking. Furthermore, the questionnaire was composed using the FSFI. Full privacy of the participants was ensured. The questions were based on their sexual performance before contracting COVID-19. The FSFI is a validated questionnaire that comprises 19 questions. The scoring scale is designed in a way that the first two questions range from one to five while the rest of the questions range from zero to five. A total of each score is calculated. The higher is an indication of a healthy sexual life. The score ranges between 1.2 and 36. An optimal cutoff value is 26 [11]. Patients were followed up after 60 days and FSFI was re-assessed. Those who could not come to the hospital physically were contacted via phone. We lost 63 participants to follow-up.

Statistical analysis was done using the Statistical Package for the Social Sciences (SPSS) v. 22.0 (IBM Corporation, Armonk, New York, United States). Mean and standard deviation were calculated for continuous variables, whereas categorical variables were presented as percentages and frequencies. The chi-square test and dependent t-test were applied to compare categorical data before and after COVID-19. A p-value of less than 0.05 meant that there is a significant difference in the value between the two groups and the null hypothesis is void.

## Results

The mean age of the participants was 39 ± 8 years and the mean number of days the participants were hospitalized was 3.1 ± 1.2 days. C-reactive protein at discharge was 10.2 ± 3.2 mg/L (Table 1).

**Table 1:** Characteristics of participants at the time of discharge CRP: C-reactive protein; IU: international unit; LDH: lactate dehydrogenase; ESR: erythrocyte sedimentation rate; SD: standard deviation

Characteristics at the time of discharge	Mean ± SD (n = 237)
Age (in years)	39 ± 8
Mean number of days at the hospital	3.1 ± 1.2
CRP (mg/L)	10.2 ± 3.2
LDH (IU)	315.6 ± 89.2
ESR (mm/h)	11.4 ± 4.3

The mean FSFI score for participants before COVID-19 was significantly higher compared to the score on follow-up (28.16 ± 1.9 vs. 24.43 ± 2.5; p-value: <0.0001). Participants who had FSFI score more than 26 were significantly higher before COVID-19 (72.5% vs. 51.0%; p-value: <0.0001) (Table 2).

**Table 2:** FSFI score of participants before COVID-19 and on follow-up COVID-19: coronavirus disease-2019; FSFI: female sexual function index; SD: standard deviation

FSFI score	Pre-COVID-19	60 days after discharge	p-Value
Total sexual score (mean ± SD)	28.16 ± 1.9	24.43 ± 2.5	<0.0001
Participants with scores of more than 26	172 (72.5%)	121 (51.0%)	<0.0001

## Discussion

Our study concluded that the mean FSFI scores were reported to be higher in women before COVID-19 compared to their scores 60 days after being discharged, i.e. 28.16 ± 1.9 and 24.43 ± 2.5, respectively. In concordance with this finding, an Italian study also obtained similar scores: 29.2 ± 4.2 before and 19.2 ± 3.3 after the disease [5]. On the other hand, a similar study in Turkey reported no significant difference in FSFI scores before (24.75 ± 6.55) and after (23.03 ± 7.87) COVID-19. The explanation stated for this difference was that they might not be feeling well, or due to less interest in sexual intercourse [11]. Our study also found that the number of patients with FSFI scores of more than 26 was considerably higher before COVID-19. It is important to note that the current pandemic has an impact on the sexual function of females, even those who have not contracted COVID-19. Fuchs et al. stated that during pandemic there was a decrease in quality of sexual lifestyle and frequency of intercourse [12]. Even though males were not included in our study, Sansone et al. pointed out that various factors may be responsible for erectile dysfunction in COVID-19, including endothelial dysfunction, stress associated with COVID-19, and sub-clinical hypothyroidism [13].

Various factors may be responsible for the decline in sexual function on COVID-19. It is a matter of fact that in stressful situations, the lifestyle is unavoidably changed. It is believed that social limitations and not knowing what would happen in the future tend to have an effect on people’s lifestyle and sexual function [14]. Increased stress, depression, anxiety, and dissatisfaction are known to significantly affect mental health [15,16]. However, the decline in the frequency of sexual intercourse secondary to stress depends on certain factors [17,18]. In such times, individuals’ priorities are changed, and sexual arousal and relationship status are certainly affected by stressful situations. As a result, women are more likely to show less sexual activity during this time span. Moreover, if the stressful time frame lasts for a long time, it may significantly affect the marital life [19,20]. Due to these factors, the overall quality of life is considerably affected.

Our study has shown a significant link between sexual function and COVID-19 in women. However, the study has some limitations as well; the sample size was small as the study was conducted in female COVID-19 unit; hence, it was less diverse and only included female. Another limit was that the participants who could not come for a follow-up were contacted via telephonic interviews, which could potentially make disposition to the outcomes. Finally, there were no data regarding the spouse of the participants getting COVID-19. Therefore, future studies involving more participants are needed to confirm the findings of our study and to cover the factors that have not been assessed.

## Conclusions

There is a significant decline in sexual function of females, who had contracted COVID-19 infection. COVID-19 survivors should be counseled properly about the impact on the sexual function when discussing long-term complications of COVID-19. Further large-scale studies, involving spouses, as well should be conducted to identify the factors associated with decline in sexual function.
